# Clinical aspects of myocardial fibrosis in adults with Ebstein’s anomaly

**DOI:** 10.1007/s00380-018-1141-5

**Published:** 2018-02-21

**Authors:** Aleksandra Ciepłucha, Olga Trojnarska, Anna Kociemba, Magdalena Łanocha, Mikolaj Barczynski, Szymon Rozmiarek, Lucyna Kramer, Malgorzata Pyda

**Affiliations:** 1 Department of Cardiology, University of Medical Sciences, ½ Dluga Street, 61-848 Poznan, Poland; 2 Cardiac Magnetic Resonance Unit, University of Medical Sciences, Poznan, Poland; 3 Department of Computer Sciences and Biostatistics, University of Medical Sciences, Poznan, Poland

**Keywords:** Ebstein’s anomaly, Congenital heart disease, Late gadolinium enhancement, Exercise capacity, Myocardial fibrosis

## Abstract

Heart failure and arrhythmia are common complications in adults with Ebstein’s anomaly. They may result not only from hemodynamic alterations, but also from myocardial fibrosis. Late gadolinium enhancement (LGE) by CMR enables the evaluation of myocardial fibrosis. The aim of the study was to asses the presence of LGE and its relation to clinical outcome. We studied a group of 37 unoperated adults aged 43.0 ± 14.4 years with Ebstein’s anomaly from the congenital heart disease outpatient clinic. Study protocol included: cardiopulmonary test, assessment of supraventricular arrhythmia (SVA), and CMR with evaluation of cardiac chambers’ morphology and function, and presence of LGE. Variables following normal distribution were shown as mean ± SD if otherwise median (range) was applied. Fibrosis was found in 18 patients (48.6%) and was distributed as follows: 12 patients (32.4%) in the right atrium, 12 (32.4%) in the atrialized right ventricle, and 2 (5.4%) in the functional right ventricle. In patients with fibrosis, the tricuspid regurgitation fraction was bigger (48.3 ± 19.7 vs. 36.1 ± 22.6%, *p* = 0.048) and SVA was more frequent [12 (66.7%) vs. 6 (31.6%), *p* = 0.046] when compared to patients without fibrosis. However, exercise capacity did not differ between patients with and without LGE (peak *V*O_2_ 24.0 ± 4.7 vs. 23.7 ± 4.4, *p* = 0.87). In adults with Ebstein’s anomaly fibrosis estimated by LGE-CMR was localized in the right atrium and the right ventricle only. Volume overload resulting from tricuspid regurgitation might be a factor conducive to fibrosis. Myocardial fibrosis did not influence exercise capacity. Association between myocardial fibrosis and supraventricular arrhythmia was confirmed.

## Introduction

Ebstein’s anomaly (EA) is an anomaly comprising 0.5% of all congenital heart diseases (CHD) in adults. This defect is characterized by a displacement of septal and posterior tricuspid leaflets toward the apex of the right ventricle (RV). As a result, the inlet portion of RV creates the “atrialized” part and functions as an atrium, whereas the trabecular part and the outflow tract constitute the functional right ventricle (fRV). These anatomical abnormalities cause a marked dilatation of the right atrium, which additionally may be influenced by a significant tricuspid regurgitation [1].

Despite the altered cardiac hemodynamics, even patients with a severe defect often reach adulthood, although their lifespan is significantly shortened. The cause of 20% of deaths is arrhythmia, whereas 50% of the population dies of heart failure [2]. It is also known that arrhythmia might be a reason as well as a result of heart failure [3]. As has been recently proven, an underlying cause of the pathophysiological phenomena mentioned above can be myocardial fibrosis [3, 4]. This process is initiated by a precipitating insult such as pressure or volume overload, ischemia, inflammation, cyanosis, or genetic predisposition [5, 6]. These stimuli activate myofibroblasts which regulate the turnover of the main extracellular protein, i.e., collagen, by synthesizing both its precursors as well as metalloproteinase responsible for collagen degradation. Specific types of collagen determine myocardial elasticity and stiffness [4]. Accumulation of these proteins has been found in histopathological examination in patients with Ebstein’s anomaly [7].

Cardiac magnetic resonance (CMR) is increasingly used to detect pathologic fibrosis and has an expanding role in noninvasive diagnosis of congenital heart diseases. Late gadolinium enhancement (LGE) estimated by CMR allows us to evaluate the presence of myocardial inflammation, fibrosis, and injury. This method is based on relative accumulation of gadolinium under these conditions as the result of slower washout kinetics and the increased extracellular volume. Clinical usefulness of this method has been demonstrated in patients with both ischemic and non-ischemic heart failure [5, 8]. In addition, the presence of fibrosis and its clinical implication has also been found in a population with congenital heart disease (CHD). Babu-Narayan et al. described fibrosis in patients with repaired tetralogy of Fallot [9], transposition of the great arteries [10], the Eisenmenger syndrome [11], and in patients after the Fontan operation [12]. To the best of our knowledge, except for one case report [13], there has been no publication addressing the issue of LGE and its pathophysiological role in the population with Ebstein’s anomaly so far.

The aim of the study was to assess the presence of myocardial fibrosis with LGE-CMR, and to determine factors leading to this process, its potential impact on exercise capacity and the occurrence of supraventricular arrhythmia in adult patients with Ebstein’s anomaly.

## Materials and methods

### Study group

A database of grown-up congenital heart disease outpatient clinic of the Department of Cardiology, University of Medical Sciences in Poznan, Poland identified 52 patients with Ebstein’s anomaly. Four of those refused to participate in the study, 5 were excluded due to the previous cardiosurgery, 5 due to implanted pacemakers, and one due to claustrophobia. The remaining 37 patients (23 males) formed our study group, aged 18–76 years. Sinus rhythm was present in all of them at the time of examination. Coexisting cardiac anomaly was present in three patients (8.1%): 2 trivial atrial septal defects and 1 mild pulmonary stenosis. The study protocol comprised the following: medical history, clinical examination, laboratory tests, 12-channel ECG, echocardiography, cardiopulmonary exercise test, and CMR.

### Cardiac magnetic resonance

**Fig. 1:**
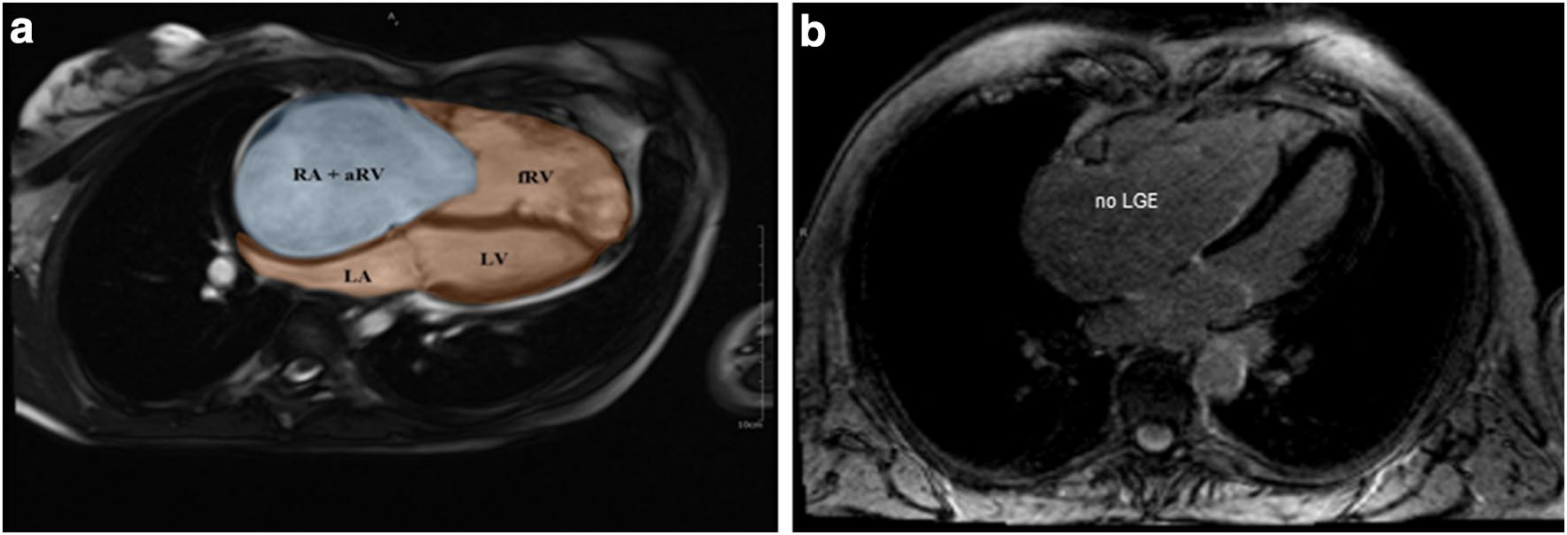
Cardiac magnetic resonance in patients with Ebstein’s anomaly. **a** End-diastolic contours of the cardiac chambers: left ventricle (LV), left atrium (LA), functional right ventricle (fRV), and functional right atrium calculated as a sum of atrialized right ventricle (aRV) and anatomical right atrium (RA). Severity index labeled Celermajer index (cmr-Cel) as the ratio of the summed volumes of RA and aRV to that of the summed volumes of fRV, LV, and LA. **b** Example of a patient with Ebstein’s anomaly without late gadolinium enhancement in the myocardium

Cardiac magnetic resonance was performed on 1.5 T scanner (Magnetom Avanto, Siemens) with the use of a six-element matrix coil combined with a 2–4 element spinal coil. All images were ECG gated and performed during patient expiration breath hold. Total examination time was 45–55 min. Steady-state free-precession imaging was used for volume assessment of the ventricles and atria. Standard 4- and 2-chamber planes with a stack of short-axis views and contiguous axial slices covering the heart from the RV outflow tract to the diaphragmatic surface of the right ventricle were used. Typical slice thickness was 8 mm without any gap. To fully characterize both, the left and right cardiac chambers the following volumes: left atrium (LA), left ventricle (LV), functional right ventricle (fRV), atrialized right ventricle (aRV), right atrium (RA), and functional right atrium (fRA) calculated as a sum of RA and aRV (Fig. 1a).

Maximal volumes of atria, right and left ventricles were analyzed (end-diastolic, end-systolic volumes of ventricles and atria, atrialized right ventricle as well as the functional right atrium). Parameters characterizing right-heart chambers were defined according to Yalonetsky et al. [14]. A line connecting the free wall attachment of the anterior tricuspid valve leaflet and the tricuspid valve annulus (i.e., the point of presumed septal leaflet attachment) was considered as the border between RA and aRV. A line connecting the free wall attachment of the anterior tricuspid valve leaflet and the septal attachment of the displaced leaflet demarcated the border between aRV and fRV. Apical displacement of the septal leaflet was measured in ventricular diastole from the 4-chamber view. The end-diastolic volume (EDV) and end-systolic volume (ESV) of the right atrium and ventricle were measured on axial and short-axis views. For the left ventricle and atrium only parameters from short axis, slices were calculated. Stroke volume (SV) was designated as the difference between EDV and ESV, the ejection fraction was calculated as SV divided by EDV. All volumes were measured using commercially available software (QMass, Medis, Leiden, The Netherlands). The tricuspid valve regurgitant volume (ml/beat) and fraction (%) were calculated from RV stroke volume (RVSV) and phase contrast velocity mapping flow measurements of the pulmonary artery according to the following formulas:

$$ {\text{Regurgitant volume (ml}}/{\text{beat)}} = {\text{RVSV (ml}}/{\text{beat)}} - {\text{pulmonary forward flow (ml}}/{\text{beat)}} . $$

$$ {\text{Regurgitant fraction (}}\% ) = {\text{regurgitant volume (ml}}/{\text{beat)}} \times 100/{\text{RVSV (ml}}/{\text{beat)}}. $$

Based on previously published studies, tricuspid regurgitation (TR) was considered severe if the regurgitant fraction exceeded 35% [15].

An index originally introduced in echocardiography by Celermajer [16] for Ebstein’s anomaly severity assessment was modified in our analysis and adjusted for CMR-derived end-diastolic volumes. It was labeled cmr-Cel and calculated as the ratio of the summed volumes of RA and aRV to that of the summed volumes of fRV, LV and LA in a 4-chamber view (Fig. 1a) [16, 17].

**Fig. 2:**
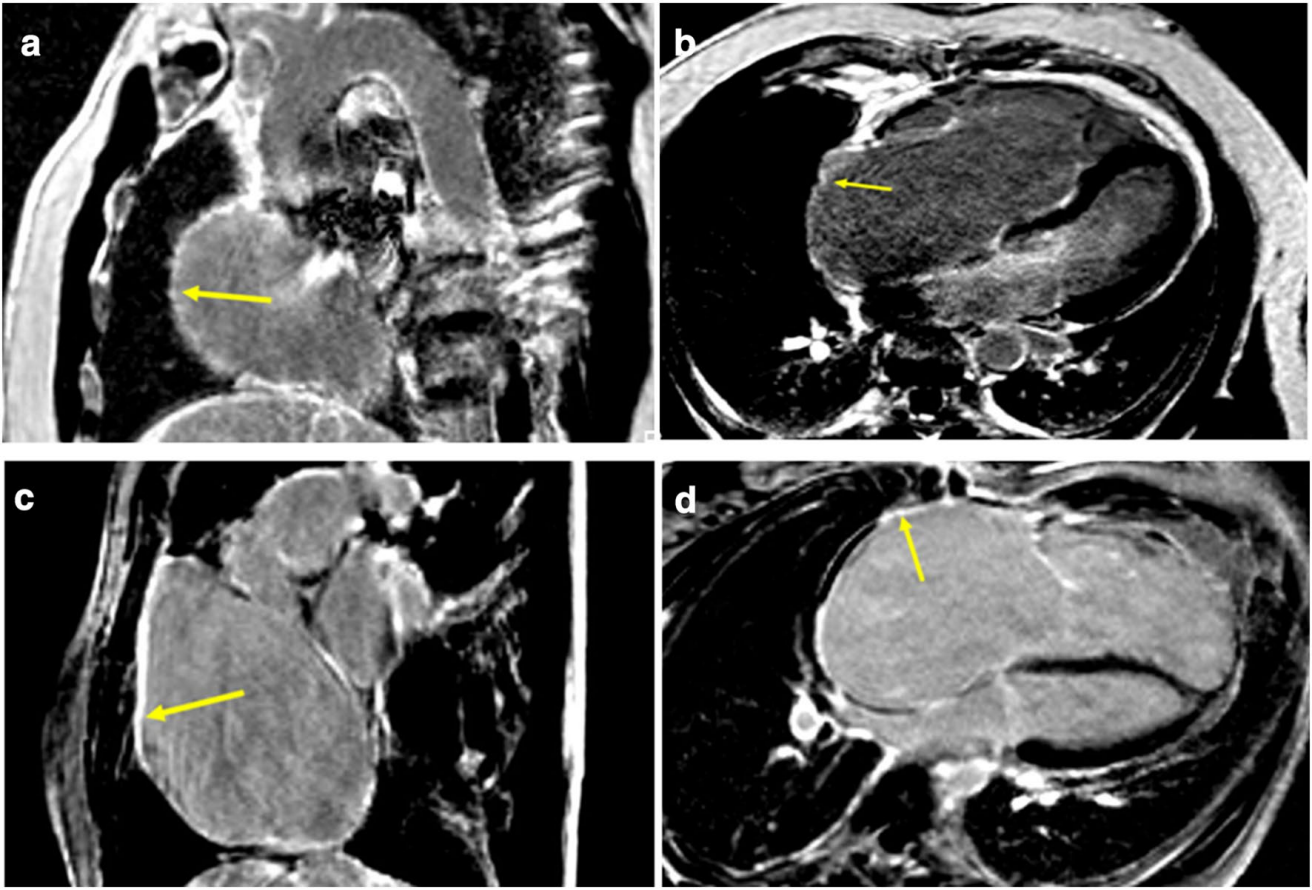
Cardiac magnetic resonance in patients with Ebstein’s anomaly. Patient with LGE localized in the functional right atrium in the short-axis view (**a**) and the four-chamber view (**b**). Another example of the patient with LGE localized in the functional right atrium in the short-axis view (**c**) and the four-chamber view (**d**); yellow arrows indicate LGE

**Fig. 3:**
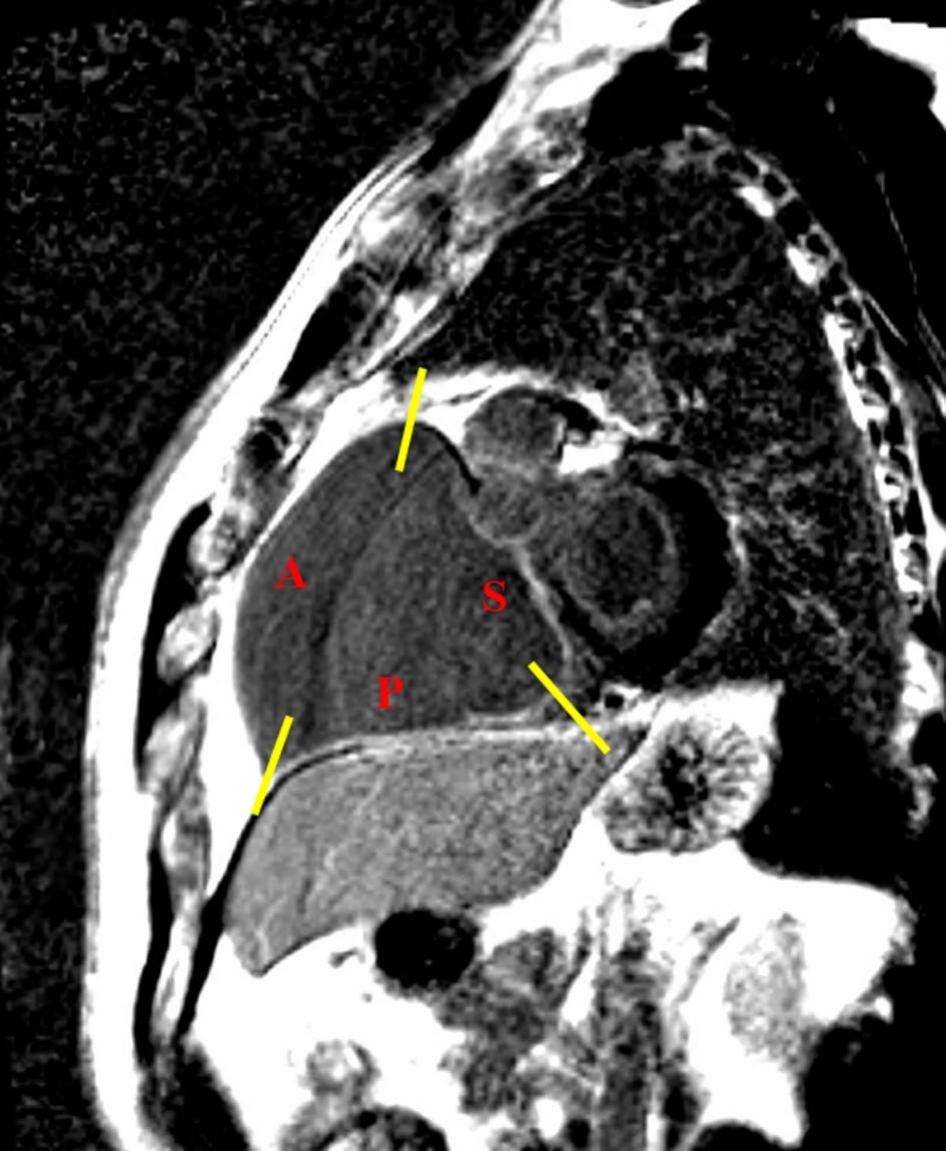
Cardiac magnetic resonance in patients with Ebstein’s anomaly. A schematic division of the functional right atrium into the three main regions. *A* anterolateral wall, *S* septal wall, *P* posterior wall

Late gadolinium enhancement was performed 10–15 min after contrast injection (0.1 mmol/kg Gadovist, Bayern). The inversion time was determined for each patient individually for complete nullification of the myocardium signal to accommodate the gradual washout of the contrast agent from the myocardium. A standard turbo flash, breath hold, two-dimensional, T1-weighted inversion recovery sequence with non-selective 180˚ inversion preparation pulse was used. The slice thickness was 8 mm, no gap, pixel size 2 × 1.5 mm, repetition time—793 ms, echo time—3.17 ms, flip angle—25°, matrix—156 × 256. Signal-to-noise ratio (SNR) was measured as a mean signal value of tissue divided by standard deviation of the image background: SNR fib = 33.25 (for fibrous tissue), SNR norm = 10.7 (for normal tissue). Contrast-to-noise ratio (CNR) was measured according to the following formula: CNR = SNR fib − SNR norm = 22.55, i.e., SNR of fibrous atrium minus SNR of normal atrium. LGE was performed in 2, 3, and 4 chambers and short-axis planes which also covered both atria. Total LGE was evaluated qualitatively as LGE present/absent and determined by location within fRV, aRV, RA, fRA, LV, and LA (Fig. 2a–d). Fibrosis was defined when signal intensity exceeded the mean signal intensity of a normal atrial wall plus two standard deviations. In addition, the signal intensity of the fibrosis had to exceed the intensity of the intra-ventricular blood signal. An inversion time between 280 and 320 ms in most cases provided successful nulling of the myocardium (there was no need for adjustment of inversion time to nullify atrial wall signal). During the acquisition, any artifacts were excluded by comparing the LGE image with an SSFP cine image taken at the same slice position. In addition, a second imaging plane was prescribed directly through the area of interest for further clarification. Atrial fibrosis was evaluated on the basis of the short-axis images. Due to distorted anatomy of the functional RA, atrial walls were divided into three main regions: septal, posterior, and anterolateral (Fig. 3). Septal region extended from the site of attachment of septal tricuspid leaflet upwards, encompassing the proper intra-atrial septum. Posterior wall of the functional RA extended upwards from the site of attachment of posterior tricuspid leaflet and encompassed the smooth portion of RA with orifices of inferior vena cava and coronary sinus; upper border of posterior wall was delineated at the level of superior vena cava orifice. Anterolateral wall extended upwards from anterior tricuspid leaflet and comprised the remaining trabecular atrial wall, being confined by the intra-atrial septum at the left border. LGE was assessed within these walls qualitatively as present or absent by two independent, experienced observers. The intraobserver agreement was 0.88, where there were no compliant results, a third observer was consulted for expertise.

### Cardiopulmonary test

The cardiopulmonary exercise treadmill test (CPET) according to the Bruce protocol and symptom limited was performed in all studied patients. The equipment was calibrated with a standard gas mixture before each test. A standard 12-lead electrocardiography was continuously recorded. Patients were encouraged to continue the exercise until the respiratory exchange ratio (RER) exceeded 1. Oxygen uptake (Peak *V*O_2_), carbon dioxide production (*V*CO_2_), and minute ventilation were measured with breath by breath technique using Sensor Medics, model *V*_max_ 29. Peak *V*O_2_ was defined as the average for the last 20 s of exercise and expressed as ml/kg/min, as well as the percentage ranges predicted for sex, age, height and weight [18]. Impaired exercise capacity was defined as %peak *V*O_2_ < 85% of predicted value [19]. A ventilation/carbon dioxide slope (*V*E/*V*CO_2_ slope) was assessed by linear regression for the whole exercise; *V*E/*V*CO_2_ slope exceeding 34 was considered as determinant of poor prognosis [20].

### Supraventricular arrhythmia

Patients’ medical data were analyzed with regard to the presence of supraventricular arrhythmia (SVA). SVA was defined as atrial fibrillation or any form of supraventricular tachycardia that originated within the tissue of His bundle or above [21]. Types encountered in Ebstein’s anomaly were as follows: focal atrial tachycardia (focal AT); macro-reentrant atrial tachycardia (reentrant AT), which included typical atrial flutter and intra-atrial reentrant tachycardia [22]; and atrial fibrillation (AF) [3, 21, 22]. Patients with a documented accessory pathway mediated tachycardia which included Wollf–Parkinson–White syndrome were not further analyzed since the substrate for this arrhythmia is proved not to be associated with the regions of fibrosis [21].

### Other parameters

Serum brain natriuretic peptide (BNP) level was measured in the venous blood samples taken after 15 min of rest in supine position using the Abbott AxSYM Immunoassay system. Functional status was assessed according to the New York Heart Association (NYHA) classification. Pulse oximetry was used to measure resting blood oxygen saturation (SO_2_). Values of SO_2_ > 95% were considered as normal.

### Statistical analysis

Data are presented as the mean value with standard deviation (for normal distribution) and median with range (non-normal distribution). For variables following Gaussian distribution, a statistical analysis was performed using the Student *t* test for unpaired samples. For variables not following normal distribution, the Mann–Whitney *U* test was used. Categorical variables presented as count and percent value were compared using the Chi-square test with Yates’ correction or Fischer Exact test. For comparisons among four groups of index ranges, an ANOVA test with post hoc Scheffe test was used following Shapiro–Wilk test for normality and Leven’s test for homogeneity of variance.

Factors associated with the presence of LGE were analyzed with univariable logistic regression. A multivariate analysis was not performed due to a small study group. Results of *p* < 0.05 were considered statistically significant. Calculations were performed using the statistical software STATISTICA v.10.

The study protocol conformed to the ethical guidelines of the 1975 Declaration of Helsinki and was approved by the local human research committee. All of the enrolled individuals gave a written informed consent to participate in the study.

## Results

Mean patients’ age was 43.0 ± 14.4 years (range 18–76). Most individuals were classified as NYHA functional class I [32 (86.5%)] or NYHA class II [5 (13.5%)]. Mean blood saturation was 97.6 ± 0.7; no case of saturation below 95% was reported. Detailed demographic and clinical characteristics of the study group are presented in Table 1.

**Table 1:** Clinical characteristics of the whole study population and with regard to the presence of myocardial fibrosis

	Study group (*n* = 37)	LGE (+) subgroup (*n* = 18)	LGE (−) subgroup (*n* = 19)	p*
Demographic and clinical features				
Age (years)	43 (18–76)	43.0 ± 17.2	40.1 ± 11.8	0.55
Male (*n*)	27 (62.8%)	13 (72.2)	8 (42.1)	0.1
BMI (kg/m^2^)	25.5 ± 4.6	25.0 ± 4.8	25.3 ± 4.4	0.85
SpO_2_ (%)	97.6 ± 0.7	97.8 ± 0.6	97.5 ± 0.7	0.89
HR (bpm)	77.9 ± 10.8	76.8 ± 9.9	78.2 ± 10.9	0.72
SBP (mmHg)	120 (90–140)	120 (110–140)	120 (90–140)	0.89
SVA (*n*, %)	18 (48.6)	12 (66.7)	6 (31.6)	0.046
Exercise capacity				
Peak *V*O_2_ (ml/kg/min)	23.4 ± 4.5	24.0 ± 4.7	23.7 ± 4.4	0.87
% peak *V*O_2_	65.5 (43.0–114.0)	65.0 (44.0–114.0)	66.0 (43.0–88.0)	0.47
*V*E/*V*CO_2_ slope	32.0 (28–47.5)	32.0 (28.4–40.0)	32.0 (28.0–47.5)	0.42
BNP (pg/ml)	51.9 (4.5–155)	65.3 (14.2–155.0)	37.6 (4.5–134.9)	0.06
Cardiac magnetic resonance				
TV displacement (mm/m^2^)	17.8 ± 6.3	17.3 ± 6.3	18.2 ± 6.4	0.703
fRV EDV index (ml/m^2^)	124.3 (52.8–378.9)	141.5 (52.8–378.9)	122.5 (77.4–267)	0.267
fRV EF (%)	40.8 ± 11.6	38.9 ± 9.8	42.7 ± 13.1	0.325
aRV EDV index (ml/m^2^)	45.9 (10.1–880.9)	45.9 (15.5–880.9)	46.7 (10.1–124.6)	0.558
RA ESV index (ml/m^2^)	154.0 ± 144.2	196.4 ± 178.0	97.7 ± 82.6	0.037
fRA EDV index (ml/m^2^)	159.5 (65.7–1571.4)	180.7 (70.8–1571.4)	122.8 (65.9–456.9)	0.065
LV EDV index (ml/m^2^)	63.7 (38.7–94.2)	67.6 (38.7–94.2)	60.4 (50.3–89.5)	0.242
LV EF (%)	57.9 ± 8.2	55.9 ± 9.2	59.9 ± 6.7	0.135
LA ESV index (ml/m^2^)	33.6 ± 8.3	35.8 ± 8.7	29.8 ± 10.3	0.035
cmr-Cel	0.7 (0.3–5.3)	0.8 (0.3–5.3)	0.6 (0.4–1.4)	0.15
TR fraction (%)	41.8 ± 20.7	48.3 ± 19.7	36.1 ± 22.6	0.048

*LGE* late gadolinium enhancement, *BMI* body mass index, SpO_2_ blood oxygen saturation, *HR* heart rate, *SBP* systolic blood pressure, *SVA* supraventricular arrhythmia excluding Wollf–Parkinson–White syndrome, *peak VO*_*2*_ peak oxygen consumption, *%peak VO*_*2*_ percentage of predicted peak *V*O_2_ values, *VE/VCO*_*2*_ *slope* ventilation/carbon dioxide slope, *BNP* brain natriuretic peptide, *fRV* functional right ventricle, *fRA* functional right atrium, *RA* right atrium, *aRV* atrialized right ventricle, *LV* left ventricle, *cmr-Cel* Celermajer index calculated in cardiac magnetic resonance, *EF* ejection fraction, *EDV index* end-diastolic volume indexed for body surface area, *EDV index* end-diastolic volume indexed for body surface area, *TV displacement* apical displacement of tricuspid septal leaflet indexed by body surface area, *TR* tricuspid regurgitation

*p value calculated for LGE (+) vs. LGE (−) subgroups

Myocardial fibrosis was found in 18 (48.6%) patients with EA, of whom 16 showed LGE in the fRA, 10 had LGE detected in RA as well as in aRV, and only 2 patients had fibrosis detected in the myocardium of fRV. Demographic and clinical characteristics of the study group are presented in Table 1.

Patients with and without LGE did not differ in the demographic data, i.e., age (43.0 ± 17.2 vs. 40.1 ± 11.8, *p* = 0.55), and sex [males: 13 (72.2%) vs. 8 (42.1%), *p* = 0.1]. We also showed no significant difference between individuals with and without fibrosis in the severity of Ebstein’s anomaly assessed by the Celermajer index [0.8 (0.3–5.3) vs. 0.6 (0.4–1.4), *p* = 0.15]. Among all CMR-derived chamber dimensions, the only significant difference between LGE (+) vs. LGE (−) patients was reported for maximal volumes of atria (RA ESV index: 196.4 ± 178.0 vs. 97.7 ± 82.6, *p* = 0.037; LA ESV index: 35.8 ± 8.7 vs. 29.8 ± 10.3, *p* = 0.035, respectively). The volume of functional RA (aRV + RA) with LGE was significantly greater when compared to the size of atria without LGE [351.0 (70.8–1571.4) vs. 279.0 (65.9–456.9) ml/m^2^, *p* = 0.037]. A significant difference in volume was also demonstrated for anatomical RA—its volume was greater in patients with fibrosis [130.6 (48.8–500) vs. 84.5 (37.5–690.5) ml/m^2^, *p* = 0.035]. Such a difference was not confirmed when analyzing the aRV volume [45.9 (17.2–91.1) vs. 46.7 (10.1–880.9), *p* = 0.54)].

**Fig. 4:**
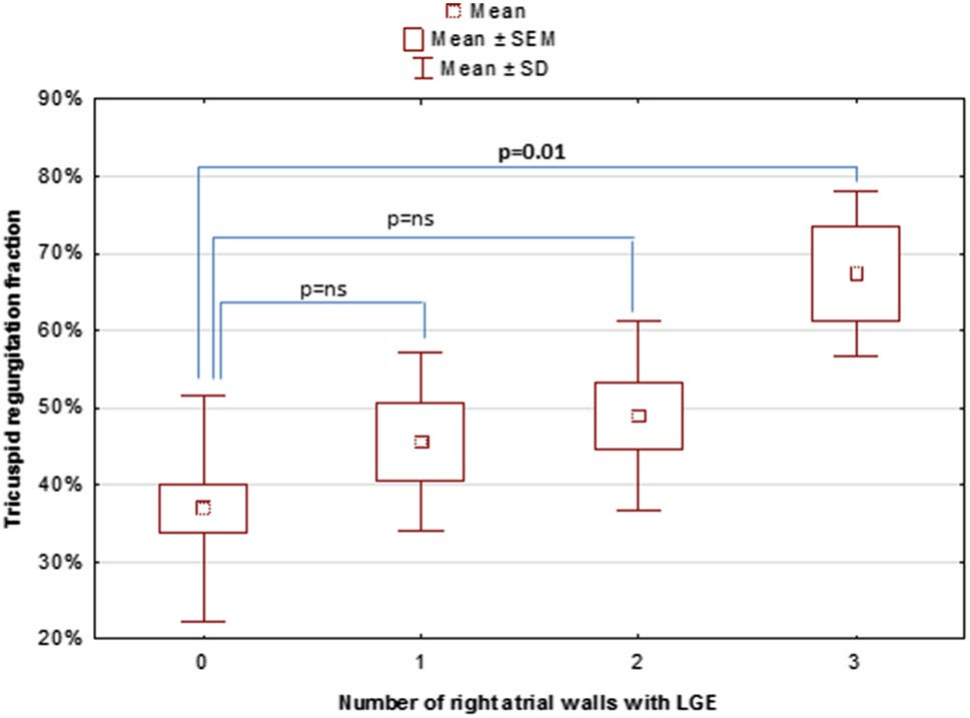
Severity of the tricuspid regurgitation fraction and the extent of myocardial fibrosis within the functional right atrium. A box plot

Severe tricuspid regurgitation was present in 26 (60.5%) individuals and regurgitation fraction was significantly greater in patients with myocardial fibrosis (48.3 ± 19.7 vs. 36.1 ± 22.6%, *p* = 0.048). A box plot (Fig. 4) showed a significant difference in the TR fraction between patients without fibrosis and those with the greatest extent of fibrosis (3 atrial walls affected) (TR fraction: 36.1 ± 14.6 vs. 67.3 ± 10.1%, *p* = 0.01). There was no significant difference in the TR fraction observed between all the other subgroups (i.e. no fibrosis vs. 1 fibrotic wall or 2 fibrotic walls; 1 fibrotic wall vs. 2 or 3 fibrotic walls; 2 vs. 3 fibrotic walls). With regard to specific atrial walls, fibrosis was seen mostly on the posterior wall [14 out of 16 (87.5%)] and on the anterolateral wall [12 out of 16 (75%)]. Septal region was affected only in 4 patients (25%) with atrial LGE.

The volume of functional RA in which fibrosis was detected was significantly greater when compared to the size of atria without LGE [351.0 (70.8–1571.4) vs. 279.0 (65.9–456.9) ml/m^2^, *p* = 0.037]. A significant difference in volume was also demonstrated for anatomical RA—its volume was greater in patients with fibrosis [130.6 (48.8–500) vs. 84.5 (37.5–690.5) ml/m^2^, *p* = 0.035]. Such a difference was not confirmed when analyzing the aRV volume [45.9 (17.2–91.1) vs. 46.7 (10.1–880.9), *p* = 0.54)].

**Fig. 5:**
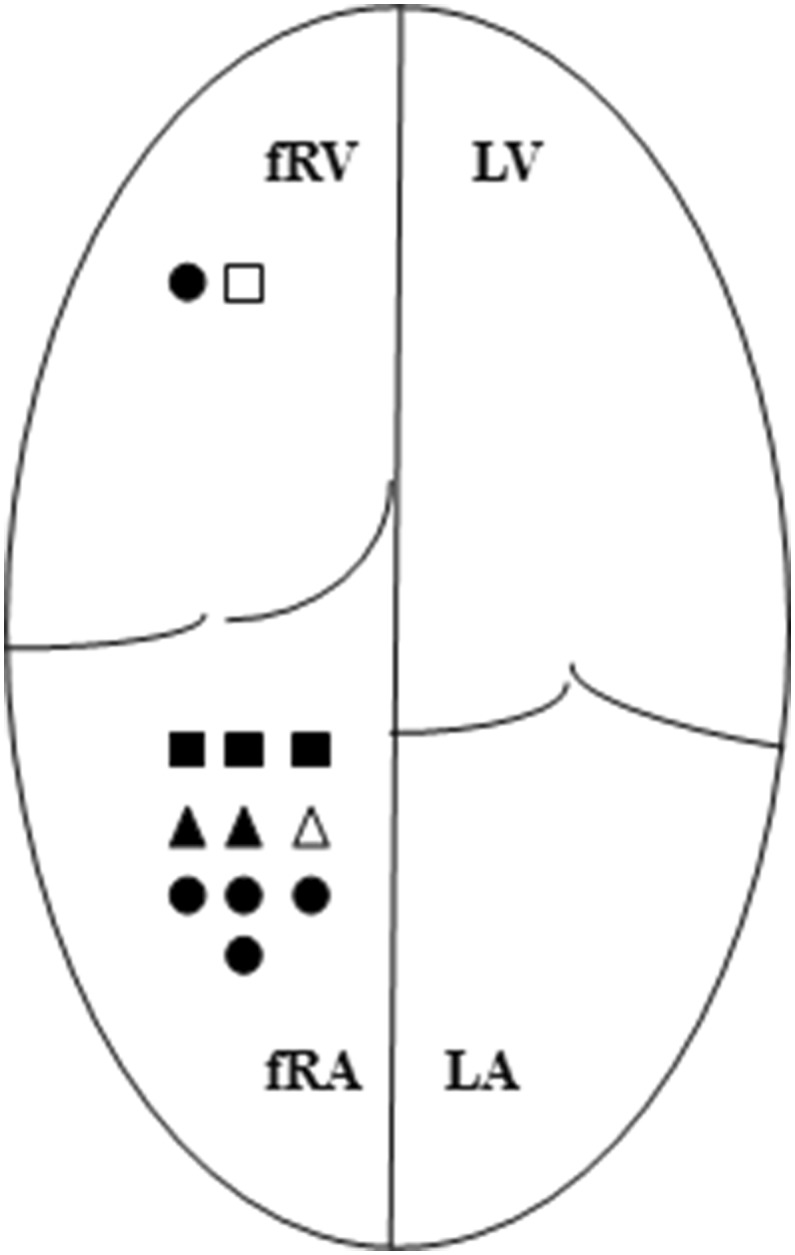
Distribution of supraventricular arrhythmia among patients with detected fibrosis. Symbols indicating arrhythmia are depicted within the chambers accordingly to the presence of fibrosis and marked white or black color depending on the severity of tricuspid regurgitation. *fRA* functional right atrium, *fRV* functional right ventricle, *LA* left atrium, *LV* left ventricle; triangle—atrial fibrillation, circle—focal atrial tachycardia, square—reentrant atrial tachycardia, black color—severe tricuspid regurgitation. *ns* not significant

Supraventricular arrhythmia was present in 18 patients (48.6%), significantly more frequently in those with LGE [12 pts (66.7%) vs. 6 pts (31.6%), *p* = 0.046]. Patients with fibrotic fRA presented SVA more frequently in than those with healthy myocardium (Fig. 5). The analysis did not show any significant relation between the presence of SVA and the occurrence of LGE in other cardiac chambers.

There were no differences in cardiopulmonary parameters and BNP concentration between patients with and without LGE—peak *V*O_2_ (24.0 ± 4.7 vs. 23.7 ± 4.4 ml/kg/min, *p* = 0.87), *V*E/*V*CO_2_ slope [32.0 (28.4–40.0) vs. 32.0 (28.0–47.5), *p* = 0.42], and BNP [65.3 (14.2–155.0) vs. 37.6 (4.5–134.9) pg/ml, *p* = 0.06].

A logistic regression analysis showed RA volume to be the only factor increasing the probability of LGE occurrence in patients with EA (OR 1.016, 95% CI 1.01–1.03, *p* = 0.049) (Table 2).

**Table 2:** Factors increasing the probability of LGE presence in patients with Ebstein’s anomaly in a univariable logistic regression analysis

Parameter	OR	95% CI	*p*
Age	1.014	0.97–1.06	0.539
Male	3.575	0.86–14.87	0.062
TR fraction	5.378	0.19–146.45	0.292
fRA ESV index	1.005	0.99–1.011	0.058
RA ESV index	1.016	1.01–1.03	0.049
aRV ESV index	1.003	0.99–1.01	0.319
fRV EDV index	1.007	0.99–1.02	0.227
cmr-Cel	3.381	0.54–21.24	0.069

*TR* tricuspid regurgitation, *fRA* functional right atrium, *RA* right atrium, *aRV* atrialized right ventricle, *fRV* functional right ventricle, *cmr-Cel* Celermajer index calculated in cardiac magnetic resonance, *ESV index* end-systolic volume indexed to body surface area, *EDV index* end-diastolic volume indexed for body surface area

## Discussion

Our analysis revealed the presence of late gadolinium enhancement in nearly half of adult patients with Ebstein’s anomaly. Myocardial fibrosis was observed only in the right atrium and right ventricle. In most cases, it was found in the functional right atrium composed of the anatomical right atrium and the atrialized right ventricle. These chambers are characterized by dissimilar histological architecture: in the wall of the anatomical right atrium, the number of cardiomyocytes is lower, whereas the amount of extracellular matrix is greater than in the right ventricle wall [23]. There are few histopathological examinations of patients with Ebstein’s anomaly available. However, the analysis of intraoperative biopsies performed by Egorov et al. confirmed that cardiomyocytes of the atrialized portion of RV preserve the specificity of ventricular myocytes and, therefore, are distinct from the atrial myocytes [24]. However, Frescura et al. [25] indicated that “the proximal atrialized ventricle often has a thinner wall than the distal functional right ventricle due to a partial absence of myocardium”. Lee et al. suggested disruption of the myofibril continuity between the functional and atrialized part of the right ventricle [26]. Therefore, it ought to be assumed that the histological structure of the atrialized RV demonstrates features in-between those that characterize the right atrium and the right ventricle.

As shown in our study, LGE occurs with the same frequency in the myocardium of the anatomical right atrium as in the atrialized right ventricle. It is, therefore, probable that the dissimilar histological structures of the right atrium and right ventricle do not determine the presence of fibrosis. It seems that it is the volume overload caused by the significant tricuspid regurgitation, which was also confirmed in the majority of our patients, that may be crucial. Another possible mechanism of fibrosis might be a mechanical insult caused directly by a regurgitant jet. However, a clear determination of underlying cause is difficult due to extremely enlarged atria and small group size. In addition, we confirmed that the severity of tricuspid insufficiency is associated with the extent of fibrosis within the functional right atrium. The end-systolic volume of the anatomical right atrium has been proven the only factor increasing the probability of fibrosis in the population studied as well. Nevertheless, there have not been many studies on LGE in the right atrium published. Sato et al. [27] reported fibrosis in 6 out of 47 (6%) patients with pulmonary hypertension; however, in this population, it was the pressure, not the volume, overload that was conducive to this pathological process.

The results of our study show that the primary insult, which in Ebstein’s anomaly patients is volume overload, seems to have only little influence on the process of fibrosis of the right ventricle. Contrarily to the atrial walls, LGE was observed within the right chamber only in two patients. Fratz et al. proved in a CMR analysis that tricuspid regurgitation determines the size of fRV. However, their study did not involve the assessment of LGE [28]. Data from other studies indicated that fibrosis in the right ventricle resulted mostly from pressure overload. These data were confirmed in a study of patients with pulmonary hypertension [29]. In addition, Sidiqui et al. [30] showed that in the population with chronic right ventricle volume overload caused by atrial septal defect, LGE was very infrequent and occurred in only 1 out of 46 patients (2%). This finding is consistent with our observation. Studies of the congenital heart diseases revealed LGE in patients after tetralogy of Fallot repair [9], atrial switch of transposition of the great arteries [10], patients with Eisenmenger syndrome [11]. It was found that the right ventricle functions under different hemodynamic circumstances and, depending on the disease progression, is overloaded either by the volume or by the pressure. It is, therefore, hard to distinguish which of these conditions is precipitating the fibrotic process.

According to current knowledge, there are several factors that can induce the process of myocardial fibrosis. They include: neurohormonal activation caused by pressure and volume overload [31, 32]), genetic factors [33], cyanosis (9), and ischemia [34]. A potential role of genetical factor among patients with Ebstein’s anomaly was proven by Postma et al. [35] who discovered an impaired gene encoding myosin—MYH7 in this population. Studies on dilated cardiomyopathy indicated that mutations in the genes encoding the cytoskeletal proteins e.g. myosin are related to familial susceptibility to myocardial fibrosis [33]. Cyanosis, common among CHD population, was shown to be one of the factors determining the presence of LGE in patients after tetralogy of Fallot repair [9]. However, it did not play a significant role among individuals with secondary pulmonary hypertension in Eisenmenger syndrome [11]. In most adults with Ebstein’s anomaly, including our study group, blood saturation was normal and can, therefore, be disregarded. Similarly, a potential impact of ischemia can be ruled out, as none of clinically significant coronary arteries abnormalities have been attributed to patients with Ebstein’s anomaly [36]. The facts discussed above justify a conclusion that it is volume overload that has a crucial impact on myocardial fibrosis pathogenesis in the study population.

The suggestion of the influence of the presence of LGE on exercise capacity was first done by Daliento et al. who demonstrated myocardial fibrosis among EA adults in autopsy examinations [7]. Contrarily to this study, we did not confirm LGE presence in the left ventricle. We have found exercise tolerance to be significantly reduced when compared to healthy population, which was also reported in other studies [20, 37]. However, our study showed no difference in exercise capacity between patients with or without LGE. This observation is in contrast with the results of the study of 92 individuals with repaired tetralogy of Fallot, which proved that fibrosis within the left and right ventricles is related to reduced exercise capacity, ventricular systolic dysfunction and increase in brain natriuretic peptide level [9]. On the other hand, no link between a widely occurring fibrosis (70% of the study group) and cardiopulmonary parameters, ventricular dimensions or systolic function was discovered in patients with Eisenmenger syndrome [11].

When analyzing the impact of fibrosis on functional status, one must consider the complex pathomechanism of exercise capacity reduction in the Ebstein’s anomaly population. The right atrium, enlarged by the atrialized part of right ventricle, causes a leftward displacement of the intra-ventricular septum. This results in a left ventricular geometry alteration, reducing its inflow and consequently cardiac output. The volume overload of the right ventricle caused by the tricuspid regurgitation is worth noting as well as is the dyssynchrony of the atrialized part which contracts during the atrial diastolic period [20, 26]. Nevertheless, in the group of patients that we studied, fibrosis was mostly limited to the functional right atrium and apparently had no significant impact on hemodynamics and consequently cardiac output. Moreover, the severity of Ebstein’s anomaly assessed by CMR with Celermajer index (originally introduced by echocardiographers) showed no relation to the presence of LGE.

Our study has proven that supraventricular arrhythmia is significantly more frequent in adults with Ebstein’s anomaly who had myocardial fibrosis confirmed by LGE. This finding is consistent with current knowledge and confirms that supraventricular arrhythmia encountered in Ebstein’s anomaly (except for Wollf–Parkinson–White syndrome resulting from the presence of accessory pathway) is thought to be an effect of enlargement and fibrosis within the functional right atrium [22]. The presence of fibrotic regions in the right atrium creates conditions for non-homogenic electrical impulse propagation which favors micro- and macro-reentrant arrhythmias to occur. Atrial fibrillation, which is a pathology typically linked to the left atrium [38], has been observed in our study group, although we found no fibrosis in this localization. However, it was reported that concerning atrial fibrillation there is a particular group of patients with a fibrotic substrate of such arrhythmia localized in the right atrium [39]. This finding has been proven in a histological examination [40]. What is also worth emphasizing is the fact that fibrosis of the atrial myocardium might be both a reason and a consequence of supraventricular arrhythmia [39], imposing a need for further research on this topic among adults with Ebstein’s anomaly. Nevertheless, we clearly confirmed the association between supraventricular arrhythmia and fibrosis within atria in patients with Ebstein’s anomaly.

### Study limitations

Our study had a cross-sectional character and was conducted on a relatively small study group. Its statistical power is, therefore, limited and the multivariate logistic regression analysis to evaluate the strongest factors related to the presence of LGE could not be done. However, other studies involving adults with congenital heart disease have carried out a comparable number of patients.

Another limitation of the study was the use of the 2D instead of 3D sequence for the assessment of atrial fibrosis. In most high spatial resolutions, atrial fibrosis is examined by a nonbreath-hold 3D sequence which is considered to be the most accurate and quickest method [41–43]. As our experience shows, 2D imaging provides better image quality than ensuring higher resolution 3D imaging. Since in this study, we were predominantly concerned with the absence or presence of fibrosis, and we believed that an extra spatial resolution could be neglected.

## Conclusions

In adults with Ebstein’s anomaly examined with CMR, late gadolinium enhancement characteristic for myocardial fibrosis was a frequent finding detected only within the right atrium and right ventricle. The volume overload resulting from tricuspid regurgitation might be conducive to the development of fibrosis. Myocardial fibrosis, localized mostly in the atria and not in the ventricles, had no negative impact on exercise capacity. However, we confirmed the association between myocardial fibrosis and supraventricular arrhythmia in this population.
